# Altered static and dynamic functional network connectivity between subcortical nuclei and cortical regions of the default mode network in type 2 diabetes mellitus

**DOI:** 10.3389/fnins.2026.1766192

**Published:** 2026-01-28

**Authors:** Dongsheng Zhang, Xiaoling Zhang, Lei Wang, Xuejiao Yan, Xiaoyan Lei, Min Tang, Jie Gao, Yarong Wang

**Affiliations:** 1 Department of Medical Imaging, The First Affiliated Hospital of Xi'an Jiaotong University, Xi'an, Shaanxi, China; 2 Department of MRI, Shaanxi Provincial People’s Hospital, Xi’an, Shaanxi, China; 3 Department of Radiology, Xi'an Daxing Hospital, Xi’an, Shaanxi, China

**Keywords:** default mode network, dynamic, fMRI, functional network connectivity, resting-state, subcortical, type 2 diabetes mellitus

## Abstract

**Introduction:**

Disruptions in functional connectivity (FC) within the default mode network (DMN) are well established as a key neuropathology underlying cognitive impairment in type 2 diabetes mellitus (T2DM). Subcortical nuclei, including the basal forebrain (BF) and mediodorsal thalamus, play critical roles in regulating DMN-associated cognitive processes and are particularly vulnerable to hyperglycemia and brain insulin resistance. However, the specific FC patterns between these subcortical nuclei and DMN cortical regions in patients with T2DM, as well as their potential associations with cognitive impairment, remain incompletely elucidated.

**Methods:**

Eighty-two patients with T2DM and 79 healthy controls (HCs) were enrolled in this study. Clinical data, neuropsychological assessments, and resting-state functional magnetic resonance imaging were collected from all participants. Resting-state (rs-FNC) and dynamic (dFNC) functional network connectivity analyses were performed to characterize connectivity between subcortical nuclei and DMN cortical regions. Correlation analyses explored associations between FNC metrics showing significant intergroup differences and participants’ clinical and cognitive parameters.

**Results:**

rs-FNC analysis revealed decreased FC between the BF and the dorsomedial prefrontal cortex (dMPFC), the BF and the temporal pole, and the dMPFC and the anteromedial prefrontal cortex in patients with T2DM (network-based statistic correction; edge *p* < 0.001, component *p* < 0.05). dFNC analyses indicated increased frequency and prolonged mean dwell time (MDT) of State 1 (high-frequency low-connectivity), as well as decreased frequency and shortened MDT of State 2 (high-frequency high-connectivity) compared with HCs (all *p* < 0.05). Reduced FC between the dMPFC and BF was positively correlated with Montreal Cognitive Assessment scores (*r* = 0.353, *p* = 0.001), whereas frequency (*r* = −0.434, *p* < 0.001) and MDT (*r* = −0.376, *p* = 0.001) of State 2 were negatively correlated with T2DM disease duration after Bonferroni correction.

**Conclusion:**

These findings indicate that T2DM duration correlates with reduced highly efficient DMN connectivity, and that the BF may regulate cognitive function via the dMPFC subsystem. The results reveal temporal and functional specificity in abnormal DMN connectivity in patients with T2DM and enrich the neural atlas of DMN dysfunction in this population.

## 1 Introduction

Type 2 diabetes mellitus (T2DM) is one of the most prevalent metabolic diseases worldwide. Multiple pathological mechanisms, such as chronic hyperglycemia, cerebral insulin resistance, and oxidative stress, induce structural and functional impairment of neurons in the central nervous system, thereby triggering cognitive decline (Ehtewish et al., 2022; Khaliq et al., 2025). Diabetes-related cognitive impairment not only leads to deficits in executive function, memory, and attention but also significantly increases the risk of progression to dementia (Sadanand et al., 2016; You et al., 2021). This condition severely impairs patients’ quality of life and imposes a heavy medical and economic burden on families and society. However, the neural mechanisms underlying T2DM-associated cognitive impairment remain incompletely elucidated.

Brain functional networks serve as the neural substrate underlying cognitive functions. Compared with functional alterations in isolated brain regions, impaired cerebral information integration capacity induced by disrupted functional connectivity (FC) between key brain regions represents a critical mechanism underpinning cognitive dysfunction (Bassett and Bullmore, 2009). The close link between the default mode network (DMN), a prominent resting-state brain functional network, and cognitive impairment in various neurodegenerative and metabolic disorders through aberrant FC is well documented (Foret et al., 2021; Wang et al., 2024; Zarifkar et al., 2021). Extensive prior research (Cui et al., 2015; Li et al., 2020; Qi et al., 2017) has confirmed structural and functional abnormalities in DMN-related brain regions of patients with T2DM from multiple dimensions. Specifically, reduced FC within the core DMN regions [posterior cingulate cortex (PCC) and medial prefrontal cortex (mPFC)] and between these core regions and other brain areas is strongly associated with declines in multiple cognitive domains, especially memory. These findings suggest that disruptions in DMN connectivity constitute the neural basis of T2DM-related cognitive impairment.

In recent years, accumulating evidence indicates that subcortical nuclei [i.e., the basal forebrain (BF) and the mediodorsal thalamus (MD)] play a crucial role as network regulatory hubs in maintaining DMN function (Alves et al., 2019; Harrison et al., 2022). Among these, the BF, a core origin of the central cholinergic system, projects many cholinergic fibers to core cortical regions of the DMN (e.g., mPFC), regulating the excitability and synchronization of cortical neurons (Gritton et al., 2016; Parikh et al., 2007). The BF is involved in DMN-mediated cognitive processes, such as learning, memory, and attention (Lozano-Montes et al., 2020), and may serve as a key node for information transmission between subcortical and cortical regions of the DMN (Aguilar and McNally, 2022; Peeters et al., 2020). The MD, a subcortical region involved in cognitive function integration, is responsible for connecting the DMN with other large-scale cortical networks and transmitting information (Greene et al., 2020), playing a unique coordinating role in advanced cognitive processes (Steward et al., 2022). More importantly, the BF is more susceptible to functional impairment induced by high glucose and brain insulin resistance than cortical regions (Izuo et al., 2023; Sposato et al., 2019), suggesting that it may be the initiating link of cognitive impairment caused by DMN dysfunction. However, the association between the FC patterns of subcortical nuclei and the DMN cortical regions, as well as cognitive function, in patients with T2DM remains unclear.

DMN, the most stable brain network in the resting state, can be subdivided into three functionally heterogeneous subsystems (Andrews-Hanna et al., 2010). The core DMN subsystem comprises the PCC and the anterior mPFC (aMPFC) and is primarily responsible for self-referential processes, acting as a hub that connects all three subsystems. The dorsomedial prefrontal cortex (dMPFC) subsystem is mainly involved in theory of mind and social cognition, whereas the medial temporal lobe (MTL) subsystem plays a critical role in autobiographical memory processes and the generation of novel thought combinations (Andrews-Hanna et al., 2010, 2014). Several previous studies have demonstrated that disrupted FC within DMN subsystems is closely associated with disease-specific clinical manifestations in various disorders, including cognitive impairment (Qi et al., 2018; Yang et al., 2023), depression (Liu et al., 2022), and substance dependence (Zhang et al., 2024). Given that subcortical nuclei serve as important regulatory nodes of the DMN, alterations in their FC with the various DMN subsystems may affect their function through specific pathways, leading to impairments in distinct cognitive domains in patients with T2DM. Therefore, unraveling the FC patterns between subcortical nuclei and the DMN subsystems could help identify critical pathways underlying subcortical–cortical dysregulation of the DMN in patients with T2DM.

Resting-state functional network connectivity (rs-FNC) has emerged as a key method for exploring the average FC strength across multiple brain regions over a given period. However, FNC exhibits dynamic fluctuations on timescales of seconds or less. This dynamic FNC (dFNC) is considered the neural basis for the brain’s flexible adaptation to the external environment and the implementation of complex cognitive functions (Cohen, 2018; Hutchison et al., 2013a). Compared with rs-FNC, dFNC is more sensitive to capturing transient changes in the brain. Therefore, the combined use of rs-FNC and dFNC methods can reveal the steady-state characteristics of brain network FC and capture its dynamic changes, enabling comprehensive multidimensional analysis of abnormal FC between subcortical nuclei and the DMN in patients with T2DM and providing abundant evidence for in-depth exploration of disease mechanisms.

The present study aimed to comprehensively characterize static and dynamic FNC alterations between subcortical nuclei and DMN cortical regions in patients with T2DM using resting-state functional magnetic resonance imaging (rs-fMRI). We further explored correlations between multidimensional FNC metrics and cognitive impairment to elucidate the mechanism by which DMN subcortical–cortical circuits modulate cognitive function in T2DM. This study provides novel directions and abundant neuroimaging evidence for clarifying the neural circuitry underlying cognitive impairment in T2DM and offers new targets for the subsequent development of drugs to delay T2DM-related cognitive decline.

## 2 Materials and methods

### 2.1 Participants

This study enrolled 85 patients with T2DM treated at the Department of Endocrinology, Shaanxi Provincial People’s Hospital between May 2023 and February 2025. We also recruited 80 healthy controls (HCs) from the hospital’s Health Examination Center through advertisements. All participants were 45–70 years old, right-handed, had ≥ 6 years of education, and a Mini-Mental State Examination (MMSE) score ≥ 24.

The inclusion criteria for the HC group were as follows: no symptoms of T2DM; fasting blood glucose (FBG) < 7.0 mmol/L; and glycated hemoglobin (HbA1c) < 6.0%. T2DM was diagnosed according to the 2014 diagnostic criteria of the American Diabetes Association. The recruited patients were without a history of hypoglycemia (blood glucose concentration <3.9 mmol/L) or hyperglycemia (blood glucose concentration > 33.3 mmol/L) and were under stable treatment (dietary control, oral medications, and/or insulin therapy).

The exclusion criteria for all participants (T2DM and HCs) were as follows: (1) inability to complete MRI examinations or unsatisfactory MRI image quality; (2) neurological disorders (e.g., Parkinson’s disease, epilepsy, cerebral infarction, brain tumor, traumatic brain injury); (3) severe psychiatric disorders (e.g., major depression, schizophrenia); (4) illicit substance abuse or alcohol abuse; (5) systemic diseases unrelated to diabetes that affect cognitive function; (6) periventricular and/or deep white matter hyperintensities (WMH) with a Fazekas score > 1 on T2-weighted fluid-attenuated inversion recovery (FLAIR) images.

The present study was reviewed and approved by the Ethics Committee of Shaanxi Provincial People’s Hospital (the ethical committee protocol number: 2022K101), and written informed consent was obtained from all participants prior to enrollment. All experimental procedures adhered to the ethical guidelines and regulations outlined in the Declaration of Helsinki.

### 2.2 Clinical data and neuropsychological tests

Medical history and clinical data were collected through medical record retrieval and questionnaires, covering indicators such as blood pressure, height, weight, and body mass index (BMI). The levels of HbA1c, FBG, triglycerides (TG), total cholesterol (TC), and low-density lipoprotein cholesterol (LDL-C) were determined via standard laboratory tests.

Multiple neuropsychological tests were used to assess the participants’ mental status and cognitive function: the MMSE and Montreal Cognitive Assessment (MoCA) evaluated global cognitive function; Parts 1 and 2 of the Color Trails Test (CTT-1, CTT-2) assessed attention and executive function; the Clock Drawing Test (CDT) measured visuospatial skills; the total immediate recall and delayed recall scores of the Rey Auditory Verbal Learning Test (RAVLT) assessed memory; the Symbol Digit Modalities Test (SDMT) evaluated information-processing speed; and the Beck Depression Inventory (BDI) was used to evaluate depressive symptoms. All neuropsychological tests were administered by psychiatrists trained in standardized testing procedures.

### 2.3 MRI data acquisition

MRI scans were performed using a 3.0 T MR scanner (Ingenia, Philips Healthcare, the Netherlands) equipped with a 16-channel phased-array head coil. Conventional T2-weighted and T2 FLAIR sequences were used to exclude visible brain lesions and assess white matter hyperintensities. The rs-fMRI data were collected using a gradient echo planar imaging (EPI) sequence with the following parameters: repetition time (TR) = 2,000 ms; echo time (TE) = 30 ms; flip angle (FA) = 90°; field of view (FOV) = 230 × 230 mm; matrix = 128 × 128; 34 axial slices (4 mm thickness, no interslice gap), acquired in an interleaved manner; and 200 volumes per scan. Sagittal three-dimensional T1-weighted images were obtained using a fast spoiled gradient echo sequence, with the following parameters: TR = 7.5 ms; TE = 3.5 ms; FA = 8°; FOV = 250 × 250 mm; matrix = 256 × 256; and 328 sagittal slices (0.55 mm thickness, no interslice gap). Participants were instructed to keep their eyes closed and remain awake during scanning.

### 2.4 rs-fMRI data processing

The rs-fMRI data were preprocessed using the DPABI software package^1^ (Yan et al., 2016). The preprocessing procedures were as follows: (1) The first 10 time points were discarded, followed by slice-timing correction and realignment of the remaining 190 time points. (2) Participants with head translation > 2 mm and/or rotation > 2 degrees were excluded. (3) Images were normalized to the standard Montreal Neurological Institute (MNI) space using an EPI template (resampling voxel size = 3 × 3 × 3 mm). (4) Nuisance signal regression was performed to remove 24 head-motion parameters, linear trends, cerebrospinal fluid signals, and white matter signals. (5) A temporal band-pass filter (0.01–0.08 Hz) was applied to reduce the influence of physiological noise. (6) All preprocessed functional images were spatially smoothed with a 6-mm full-width at half-maximum (FWHM) Gaussian kernel to improve signal-to-noise ratio.

### 2.5 Definition of regions of interest

Based on prior research (Andrews-Hanna et al., 2010), we divided the DMN into three subsystems and defined eleven 6-mm spherical regions of interest (ROIs) in MNI space. Specifically, the aMPFC and PCC constitute the core subsystem. The dMPFC, temporal–parietal junction (TPJ), lateral temporal cortex (LTC), and temporal pole (TempP) belong to the dMPFC subsystem. The ventral medial prefrontal cortex (vMPFC), posterior inferior parietal lobule (pIPL), retrosplenial cortex (Rsp), parahippocampal cortex (PHC), and hippocampal formation (HF) are included in the MTL subsystem.

Additionally, an ROI for the BF was generated using the probabilistic atlases embedded in the SPM Anatomy Toolbox.^2^ For the MD region, ROIs were constructed using the Automated Anatomical Labeling Atlas 3.^3^

### 2.6 Static and dynamic FNC analysis

The rs-FNC was defined as the Pearson correlation coefficient between ROI pairs. Specifically, mean time series were extracted from each ROI to construct a 13 × 13 functional connectivity matrix for each participant. Pearson correlation coefficients were computed for every ROI pair and then subjected to Fisher’s r-to-z transformation to normalize the distribution.

The dFNC was defined as the temporal variability in Pearson correlation coefficients across the scanning session. Sliding-window analysis was used to compute dynamic FC. All relevant analyses were performed using the Dynamic BC toolbox^4^ (Liao et al., 2014). Specifically, a window length of 30 TRs and a step size of 1 TR were used (Hutchison et al., 2013b), yielding 66 windowed datasets per participant. For each sliding window, Pearson correlation coefficients were computed for all ROI pairs, and the variance of these coefficients across all windows was used to characterize the dFNC strength.

K-means clustering was performed on all intra-window FNC matrices using 2–6 clusters and the standardized Euclidean distance metric. The optimal cluster number (*k* = 3) was determined in accordance with the standard clustering algorithm integrated in the dynamic BC software. The FNC matrices of all participants were assigned to these three states based on similarity to the cluster centroids, enabling evaluation of the time-recurrent FNC patterns across participants. Finally, temporal properties of dFNC states were calculated using state-transition vectors, including frequency, mean dwell time (MDT), and total transition number (TTN). All temporal properties were converted to standardized *Z*-scores for subsequent statistical analyses.

### 2.7 Statistical analysis

Statistical analysis was performed using IBM SPSS Statistics for Windows, Version 24.0 (IBM Corporation, Armonk, NY, United States). Independent-sample *t*-tests and Mann–Whitney *U* tests were used to compare intergroup differences in normally and non-normally distributed continuous variables, respectively, including demographic data, laboratory results, and neuropsychological scores. Intergroup sex differences were assessed using the chi-squared test. A *p*-value < 0.05 was considered statistically significant.

Based on the GTENTA software (Wang et al., 2015), two independent-sample *t*-tests were performed to compare rs-FNC and dFNC differences between groups, with BMI, TC, TG and LDL-C as covariates. The network-based statistic (NBS) method was used to correct for multiple comparisons, with thresholds set at the edge level (*p* < 0.001) and the component level (*p* < 0.05). With BMI, TC, TG and LDL-C as covariates, rs-FNC and dFNC indices showing significant intergroup differences were extracted, and their associations with clinical and cognitive variables were assessed using Pearson and Spearman correlation analyses, respectively.

## 3 Results

### 3.1 Clinical and neuropsychological characteristics

Four participants were excluded from the final analyses: two patients with T2DM due to excessive head motion, and two (one patient with T2DM and one HC) due to Fazekas scores > 1 on T2 FLAIR images. The final cohort included 82 patients with T2DM and 79 HCs, whose demographic, clinical, and neuropsychological characteristics are summarized in Table 1.

**Table 1:** Demographic, clinical, and neuropsychological characteristics of the participants.

Variable	HC (*n* = 79)	T2DM (*n* = 82)	*t*/*U*/*χ*^2^ value	*P*-value
Sex (*n* male/female)	43/36	52/30	1.343	0.247^#^
Age (years)	52.11 ± 8.25	53.18 ± 7.52	−0.860	0.391
Education (years)	13.26 ± 2.98	13.74 ± 2.68	−1.098	0.274
Disease duration (months)	–	85.10 ± 8.33	–	–
Systolic BP (mmHg)	121.26 ± 9.24	125.22 ± 18.05	−1.305	0.194
Diastolic BP (mmHg)	81.23 ± 9.89	80.43 ± 12.04	0.384	0.702
BMI (kg/m^2^)	23.58 ± 2.67	25.43 ± 3.49	−3.760	<0.001*
FBG (mmol/L)	5.06 ± 0.55	7.63 ± 2.56	−8.572	<0.001*
HbA1c (%)	5.56 ± 0.42	8.00 ± 2.05	−10.521	<0.001*
TG (mmol/L)	1.24 (1.08)	1.52 (1.54)	−2.244	0.025^△^*
TC (mmol/L)	4.34 ± 1.15	4.92 ± 0.96	−3.279	0.001*
LDL-C (mmol/L)	2.50 ± 0.87	2.97 ± 0.80	−3.350	0.001*
BDI	3 (4)	3 (6)	−0.984	0.325^**△**^
MMSE	28.67 ± 1.25	28.46 ± 1.06	1.130	0.260
MoCA	25.27 ± 2.67	24.25 ± 2.53	2.491	0.014*
CDT	24.96 ± 5.09	24.30 ± 5.63	0.783	0.435
CTT-1	76.50 (41.50)	76.50 (35.25)	−0.239	0.811^**△**^
CTT-2	146.00 (63.00)	160.50 (61.75)	−1.103	0.270^**△**^
RAVLT-immediate	42.59 ± 10.21	42.47 ± 9.64	0.080	0.936
RAVLT-delayed	7.95 ± 3.62	8.07 ± 3.12	0.233	0.816
SDMT	43.31 ± 11.67	40.10 ± 11.46	2.298	0.023*
Fazekas score (*n* 0/1)	22/57	17/65	1.110	0.292^#^

Normally distributed variables are presented as mean ± standard deviation; not normally distributed variables are presented as median (interquartile range). HC, healthy control; T2DM, type-2 diabetes mellitus; BP, blood pressure; BMI, body mass index; FBG, fasting blood glucose; HbA1c, glycated hemoglobin; TG, triglyceride; TC, total cholesterol; LDL-C, low-density lipoprotein cholesterol; BDI, Beck Depression Inventory; MMSE, Mini-Mental State Examination; MoCA, Montreal Cognitive Assessment; CDT, Clock-Drawing Test; CTT-1, Color Trails Test part 1; CTT-2, Color Trails Test part 2; RAVLT, Rey Auditory Verbal Learning Test; SDMT, Symbol Digit Modalities Test. *P*-values for the *t*-tests; ^#^*p*-values for χ^2^ tests; ^△^*p*-values for the Mann–Whitney U test. *p < 0.05.

FBG, HbA1c, BMI, TC, TG, and LDL-C in the T2DM group were significantly higher than in the HC group, while the MoCA and SDMT scores were significantly lower (all *p* < 0.05). The groups were statistically comparable in sex, age, educational level, systolic blood pressure, diastolic blood pressure, BDI, MMSE, CDT, CTT-1, CTT-2, RAVLT-immediate, RAVLT-delayed, and Fazekas scores (all *p* > 0.05).

### 3.2 Intergroup differences in rs-FNC and dFNC

**Figure 1:**
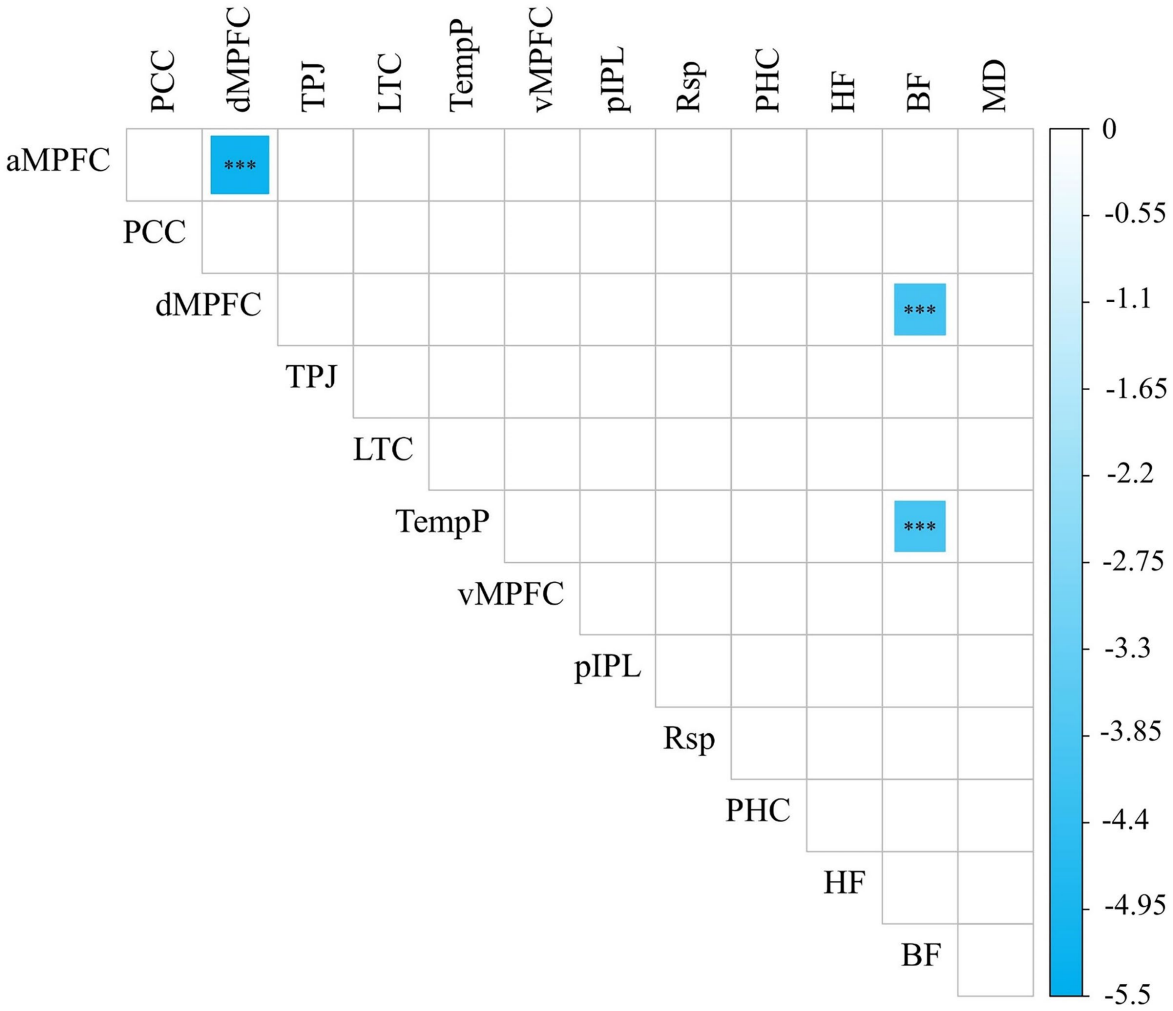
Statistically different rs-FNCs between patients with T2DM and HCs (NBS correction; edge *p* < 0.001, component *p* < 0.05). The color bar represents *t*-values, and *** denotes *p* < 0.001. rs-FNC, resting-state functional network connectivity; T2DM, type 2 diabetes mellitus; HCs, healthy controls.

After adjusting rs-FNC for BMI, TC, TG and LDL-C as covariates, patients with T2DM had lower FC between BF and dMPFC, BF and TempP, and dMPFC and aMPFC than the HC group (NBS correction; edge *p* < 0.001, component *p* < 0.05; Figure 1).

**Figure 2:**
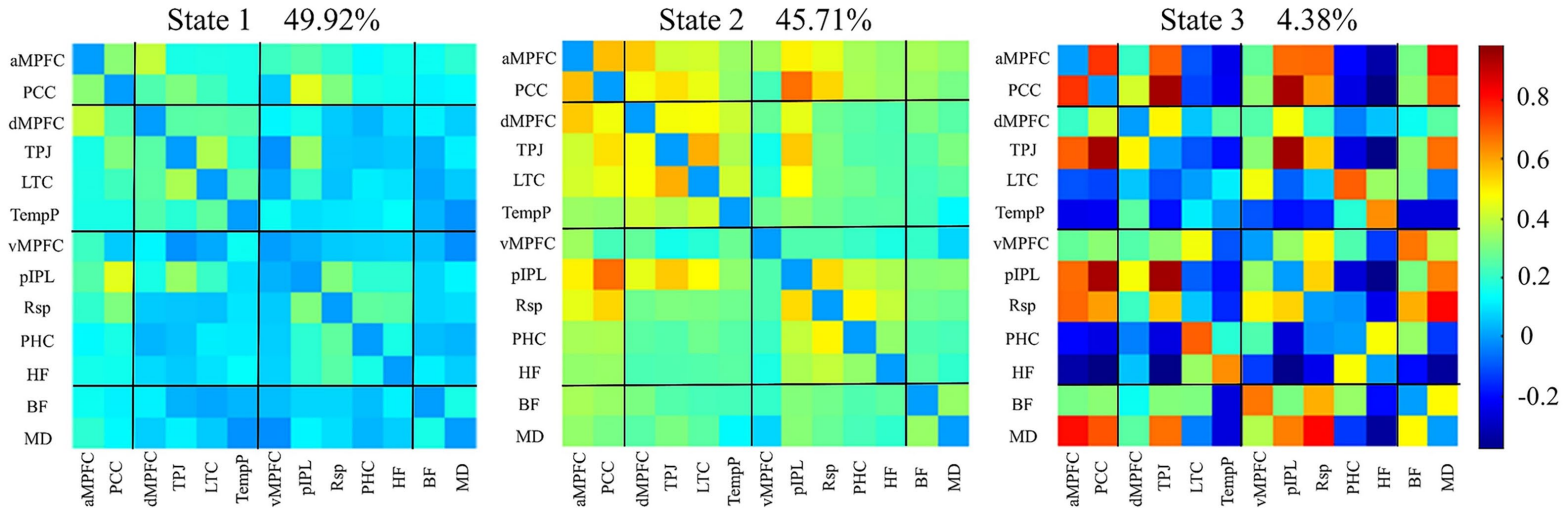
dFNC states of all subjects identified by cluster analysis. State 1 (49.92%; high frequency, low connectivity); State 2 (45.71%; high frequency, high connectivity); and State 3 (4.38%; low frequency, high connectivity). dFNC: dynamic functional network connectivity.

Cluster analysis of dFNC identified three main states in all participants (Figure 2): State 1 (high frequency, low connectivity [prolonged MDT]), State 2 (high frequency, high connectivity), and State 3 (low frequency, high connectivity). States 1 and 2 were more dominant in patients with T2DM than in HCs (all *p* < 0.05). No significant intergroup differences were observed in TTN for States 1 and 2, or in temporal properties for State 3. After adjusting for BMI, TC, TG, and LDL-C as covariates, there were no significant intergroup differences in the coefficient of variation of FC across the three States (NBS correction; edge *p* < 0.001, component *p* < 0.05).

### 3.3 Correlation between the FNC and clinical and cognitive variables

**Figure 3:**
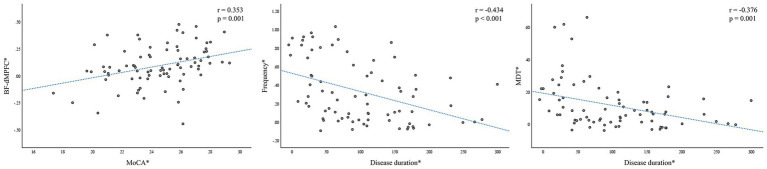
Correlations between intergroup-differentiated FNC variables and clinical/cognitive scoring variables. **(A)** FC between dMPFC and BF and MoCA scores in patients with T2DM are significantly positively correlated (*r* = 0.353, *p* = 0.001). **(B)** Significant negative correlation between the frequency in State 2 and disease duration in patients with T2DM (*r* = −0.434, *p* < 0.001); **(C)** Significant negative correlation between the MDT in State 2 and disease duration in patients with T2DM (*r* = −0.376, *p* = 0.001). The asterisk (∗) indicates coordinate values controlling for the influence of BMI values. BF, basal forebrain; dMPFC, dorsomedial prefrontal cortex; FC, functional connectivity; FNC, functional network connectivity; MDT, mean dwell time; MoCA, Montreal Cognitive Assessment; T2DM, type 2 diabetes mellitus.

After adjusting for BMI, TC, TG, and LDL-C as covariates, the decreased FC between the BF and dMPFC in patients with T2DM was positively correlated with MoCA scores (*r* = 0.353, *p* = 0.001). Furthermore, both frequency (*r* = −0.434, *p* < 0.001) and MDT (*r* = −0.376, *p* = 0.001) in State 2 were negatively correlated with disease duration in patients with T2DM. All correlations remained statistically significant after applying Bonferroni correction for multiple comparisons (Figure 3).

## 4 Discussion

This study utilized static and dynamic FNC analyses to comprehensively characterize alterations in FC between subcortical nuclei and DMN cortical regions in patients with T2DM. Compared with HCs, patients with T2DM exhibited distinct rs-FNC and dFNC patterns. rs-FNC abnormalities were primarily characterized by reduced FC between BF and cortical regions (rather than MD), indicating specificity in subcortical–cortical pathway alterations. While dFNC did not show significant subcortical–cortical connectivity abnormalities, patients with T2DM exhibited decreased frequency and shortened MDT of the DMN strong connectivity state, whereas the opposite is true for the weak connectivity state, which reflecting an abnormal temporal preference in DMN dynamic balance.

Numerous previous studies (Bloem et al., 2014; Mei et al., 2024) have confirmed the existence of a clear cholinergic fiber projection pathway between the BF and the dMPFC, which is involved in various higher cognitive functions. A study (Markello et al., 2018) in healthy populations has further identified extensive FC between the BF and the temporal lobe (including the middle and inferior temporal gyri and the hippocampus), suggesting that the BF may participate in the coordinated regulation of cognitive networks through multiple pathways. Chronic hyperglycemia and brain insulin resistance are important triggers of cholinergic pathway dysfunction (Balbaa et al., 2017; Sherin et al., 2012), and a neuroimaging study (Roy et al., 2020) has confirmed a significant reduction in BF gray matter volume in patients with T2DM. This abnormal change may be a potential pathological basis for reduced FC between the BF and the dMPFC and TempP observed in patients with T2DM in this study. Notably, both the dMPFC and the TempP belong to the dMPFC subsystem, which mediates social cognitive processes (e.g., theory of mind, emotion perception) and general cognitive functions such as executive function and memory (Andrews-Hanna et al., 2014; Muller et al., 2020; Setton et al., 2022). Although social cognition-related assessment items were not included in this study, previous research (Carlson and Moses, 2001) found a significant positive correlation between theory of mind and core dimensions of executive function (inhibitory control, cognitive flexibility, working memory), suggesting a shared dependence on the prefrontal cortex. A recent study (Oeo Morin and Keulers, 2024) has further confirmed that the accuracy of working memory in executive function is the only significant positive predictor of social cognitive theory of mind (e.g., understanding others’ complex intentions), and that the interaction between social cognition and memory is modulated by age and executive function (Chang et al., 2025). These studies collectively indicate that social cognition and general cognitive functions do not exist in isolation but have clear coordinated regulation and interdependent effects. Interestingly, this study found that the reduced FC between the BF and the dMPFC in patients with T2DM was significantly correlated with the MoCA score. This result suggests that the poor FC between the subcortical BF and the dMPFC may be the neuroimaging basis for general cognitive impairment in patients with T2DM and indirectly suggests that these patients may have abnormal social cognitive functions, consistent with existing clinical evidence of social cognitive decline in T2DM (Pisanti et al., 2023; Yasui-Furukori et al., 2019). Therefore, the observed abnormalities in BF–dMPFC FC may provide novel neuroimaging insights into the neural mechanisms underlying T2DM-related social cognitive impairment.

As a core region of its subsystem, the dMPFC also serves as a critical node mediating interactions between the DMN and external networks (Denny et al., 2012), with distinct subregions are closely connected to the DMN, dorsal attention network, and salience network (Eickhoff et al., 2016). The aMPFC is a hub of the DMN core subsystem, connecting the dMPFC and MTL subsystems to achieve information integration across cognitive domains (Andrews-Hanna et al., 2010). Reduced rs-FC between the dMPFC and the aMPFC in patients with T2DM may indicate that the information transmission pathway between the core and the dMPFC subsystems of the DMN is blocked, preventing patients from integrating internal and external information in a timely manner and leading to inappropriate judgments about current situations. As a core brain region for internal and external information exchange, mPFC dysfunction contributes to declines in attention, decision-making, and other cognitive functions (Jobson et al., 2021). Consistent with this assumption, patients with T2DM in this study showed significantly reduced SDMT scores, which may be associated with impaired mPFC-related neural pathways. Previous fMRI studies (Cui et al., 2015; Liu et al., 2019) have confirmed widespread FC disruption in the core DMN regions in patients with T2DM, which is associated with declines in various cognitive functions. The current study extends these findings by identifying reduced FC between mPFC subregions, enriching the neural map of DMN dysfunction in T2DM. Notably, no significant FC differences were observed between the MD and DMN cortical regions. This may be because the MD, a subcortical region involved in integrating cognitive function, is mainly responsible for information transmission between the DMN and other large-scale cortical networks (e.g., the executive control network) (Baxter, 2013; Zhao et al., 2024). Since this study focused on internal connectivity within the DMN and did not involve cross-network interactions, it may have missed potential abnormalities.

The dFNC results revealed no significant changes in the internal transition patterns of the DMN in patients with T2DM; however, these patients exhibited a prolonged preference for the high-frequency weak connectivity state (State 1) and reduced engagement in the high-frequency strong connectivity state (State 2). This abnormal temporal preference in DMN dynamic balance is consistent with previous findings (Xu et al., 2023). In this study, State 2 was characterized by robust intra- and inter-subsystem coupling within the DMN core and dMPFC subsystems, supporting efficient cross-subsystem integration of internal and external information and maintaining normal cognitive function (Campbell et al., 2013). In contrast, the weak FC in State 1 implies relatively low information transfer efficiency (Tian et al., 2018). Efficient information transfer is a prerequisite for the brain to complete various cognitive tasks and realize cognitive functions (Cao et al., 2021; Ito et al., 2017; Wang et al., 2021). Therefore, the results of this study suggest that the DMN of patients with T2DM is in a state of prolonged inefficient connectivity, possibly leading to low information transmission efficiency. This is consistent with the reduced SDMT score observed in patients with T2DM in this study. Correlation analysis further revealed that prolonged disease was correlated negatively with the frequency and MDT of State 2. This finding suggests that the highly efficient connectivity of the DMN gradually diminishes as the disease progresses, consistent with previous studies showing that longer disease duration was associated with more pronounced impairments across multiple cognitive domains and a significantly elevated risk of dementia in patients with T2DM (Okereke et al., 2008; Tabesh et al., 2025). Notably, no significant dFNC abnormalities were observed in subcortical nucleus–DMN cortical regions. This result may reflect the potential time-scale specificity of DMN impairment: at the transient dynamic level, changes in subcortical–cortical connectivity are still insignificant; whereas at the static dimension over longer time windows, the FC strength of this pathway is significantly decreased in patients with T2DM, a phenomenon that may originate from the persistent cumulative effect of long-term subtle dynamic damage. These findings may provide a potential biomarker for assessing the severity of T2DM-related FC disorders.

### 4.1 Limitations

This study has several limitations. First, given that the detection of molecular indicators, such as cerebral cholinergic neurotransmitter concentration, is invasive and difficult to implement in humans, this aspect was not included in this study. Therefore, direct association between abnormal subcortical–cortical FC in the DMN and impaired cholinergic pathways in patients cannot be confirmed at this time. Future targeted verifications will be conducted based on positron emission tomography studies or animal experiments to further elucidate this mechanism. Second, this study focused on the FC pattern of the DMN subcortical–cortical pathway; it did not cover other cognition-related networks such as the executive control and salience network, failing to fully reveal potential changes in the MD in cross-network regulation. Third, the cognitive assessment scales used in this study did not include social cognition-related dimensions. Subsequent studies will supplement relevant assessment tools to provide more comprehensive behavioral support for the research results. Finally, in this study, patients with T2DM were treated with multiple medications including insulin, and therefore, we could not exclude the potential effects of various medications and insulin on the study results.

## 5 Conclusion

This study systematically examined FNC characteristics between subcortical nuclei and the DMN cortex in patients with T2DM. Our findings demonstrate that T2DM duration correlates with a reduction in highly efficient connectivity within the DMN, and that BF may regulate cognitive function via the dMPFC subsystem. The inconsistency between rs-FNC and dFNC may suggest temporal and functional specificity in DMN connectivity abnormalities in patients with T2DM. This work enriches the neural map of DMN dysfunction in T2DM and offers a novel perspective for deciphering the neural mechanisms underlying disease-related cognitive impairment.

## Data Availability

The raw data supporting the conclusions of this article will be made available by the authors, without undue reservation.

## Footnotes

1
http://rfmri.org/dpabi/

2
https://www.fz-juelich.de/en/inm/inm-7/resources/jubrain-anatomy-toolbox/jubrain-toolbox

3
http://www.gin.cnrs.fr/en/tools

4
http://www.restfmri.net/forum/DynamicBC
